# Genomic Characterization of Diverse Gyroviruses Identified in the Feces of Domestic Cats

**DOI:** 10.1038/s41598-019-49955-8

**Published:** 2019-09-16

**Authors:** Jiang-Ting Niu, Shu-Shuai Yi, Guo-Ying Dong, Yan-Bing Guo, Yan-Li Zhao, Hai-Long Huang, Kai Wang, Gui-Xue Hu, Hao Dong

**Affiliations:** 1 College of Animal Science and Technology, Jilin Agricultural University, Changchun, China; 2 College of Global Change and Earth System Science, Beijing Normal University, Beijing, China; 3 Jilin Institute of Animal Husbandry and Veterinary Science, Changchun, China; 4 Library, Jilin Agricultural University, Changchun, China; 5 College of life Science, Jilin Agricultural University, Changchun, China

**Keywords:** Phylogenetics, Viral evolution

## Abstract

Gyroviruses (GyVs) are small, single-stranded, circular DNA viruses in the genus *Gyrovirus*, which consists of the chicken anemia virus (CAV) prototype and nine other viral species. These different GyV species have been reported in chickens, humans, mice, and companion animals. To date, CAV has been identified in the feces of domestic cats, while the circulation of other GyV species in cats is currently unknown. In the present study, 197 fecal samples were collected from pet cats in northeast China, and samples were screened for different GyV species by PCR. Twelve GyV strains were identified from the feces of pet cats. These included 4 positive for CAV, 3 for HGyV/AGV2, 3 for GyV3 and 2 positive for GyV6. The complete genome sequences of the 12 cat-sourced GyV strains showed 93.9–99.7% nucleotide identities to the homologous reference GyV strains. Phylogenetic analyses based on the complete genomes, VP1, VP2 and VP3 genes showed the identical classification of GyV species with previous reports. Moreover, one and four unique amino acid substitutions were identified in the VP1 protein of the cat-sourced HGyV/AGV2 and GyV6 strains, respectively, and one substitution was also observed in the VP2 protein of one GyV6 strain identified in this study. In conclusion, our investigation demonstrates that the diverse GyV species were circulating in domestic cats, and provides the first molecular evidence for the circulation of HGyV/AGV2, GyV3 and GyV6 in domestic cats. These cat-origin GyVs possessed considerable genetic diversity. This study also raises the possibility that domestic cats, as reservoirs for gyroviruses, may inadvertently disseminate viruses to other species, e.g., humans and chickens.

## Introduction

Gyrovirus (GyV) is a small, non-enveloped, icosahedral virus with a single-stranded, negative-sense, circular DNA genome, approximately 2.3-kb in length. The GyV genome contains three partial overlapping open reading frames (ORFs) encoding structural protein VP1, non-structural protein VP2 and VP3 (also named as Apoptin), respectively^1^. Of these, VP1 is the most important viral structural protein determining viral replication, infection ability and virulence^2,3^. Both VP1 and VP2 can induce neutralizing antibodies^4^. GyVs belong to the genus *Gyrovirus* originally classified in the family *Circoviridae*. Because GyVs are not either structurally or genetically related to the members of the family *Circoviridae*, the genus *Gyrovirus* was reassigned to the family *Anelloviridae* according to the recent viral taxonomy report of International Committee on Taxonomy of Virus (ICTV)^5^. To date, the *Gyrovitus* genus only includes one officially recognized species, chicken anemia virus (CAV). CAV was first isolated from bursa of Fabricius of 1-day old chickens in 1979 in Japan and had been commonly found in chickens worldwide^6,7^, leading to significant losses in the poultry industry.

In 2011, two GyVs genetically related to CAV, human gyrovirus (HGyV) and avian gyrovirus 2 (AGV2), were found in the surface of healthy French adults’ skin^8^ and in the diseased chickens from Brazil^9^, respectively. Because HGyV shared more than 93% nucleotide identities with AGV2 in VP1-VP3 gene, they are classified as the same species, named as HGyV/AGV2. Since 2011, nine novel, unofficially recognized GyV species have been identified in different hosts. HGyV/AGV2 has been identified in sera and tissues of chickens^10–12^, commercial poultry vaccines^13^, the blood of healthy blood donors^14^, HIV-infected patients and organ transplant recipients^15^, and the skin of healthy humans^8^. The third gyrovirus named GyV3 was identified for the first time in the feces of diarrhoeic children from Chile in 2012^16^ and had been also found in chickens with transmissible viral proventriculitis and human feces in China^17,18^. GyV4 was found in chicken meat and human stools in Hong Kong^18^. In 2013, GyV5 and GyV6 were first detected in the feces of Tunisian children with diarrhea^19^. Two additional species, GyV7 and GyV8 were reported to infect chicken^20^ and fulmar (sea bird)^21^, respectively. The ninth gyrovirus (GyV9) was found in the feces of a diarrhoeic adult in 2015 in the USA^22^. Recently, a newly described gyrovirus, GyV10, was identified in the sera of a crested screamer (*Chauna torquata*) with severe neurologic disease^23^. All gyrovirus species can be phylogenetically divided into three clades, A (CAV, HGyV/AGV2, GyV3, GyV6, GyV7, GyV9 and GyV10), B (GyV4 and GyV5) and C (GyV8).

Gyroviruses are not only reported to infect human and avian species, but also found in companion animals. Feher, *et al*. investigated the fecal virome of pet ferrets by metagenomic sequencing, and found the genome of CAV, HGyV/AGV2, GyV3 and GyV4 in the ferret feces^24^. Moreover, CAV had also been detected from the feces of domestic cats^25^ and dogs^26,27^ in China. These findings suggest that companion animals may be the reservoirs for gyrovirus. However, the circulation of other gyrovirus species in companion animals is currently unknown and the further investigation is required.

In this study, we investigate the prevalence of different GyV species in the feces of domestic cats in five cities in northeast China, and identify 12 cat feces-derived gyrovirus strains, including 4 CAVs, 3 HGyV/AGV2 strains, 3 GyV3 strains and 2 GyV6 strains. Moreover, the complete genome sequences of these 12 gyrovirus strains are amplified and sequenced by inverse PCR, and are also analyzed using various phylogenetic methods to describe the genomic characteristics. Based on our investigation, we demonstrate that the diverse gyrovirus species were circulating in domestic cats, and these cat-origin gyroviruses possess considerable genetic diversity.

## Results

### Epidemiology of gyrovirus in domestic cats

**Figure 1:**
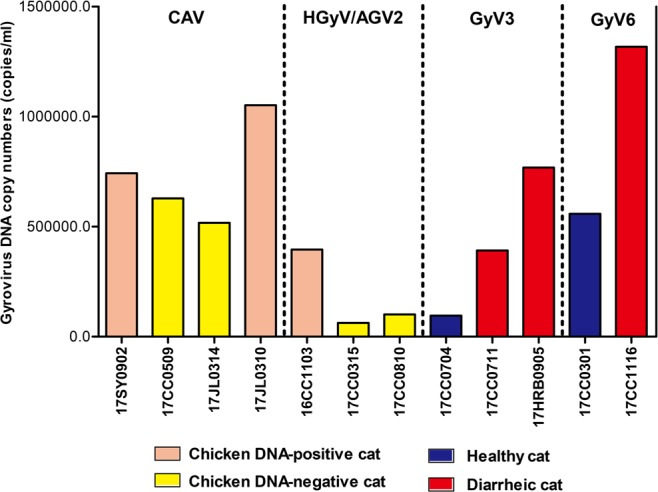
Comparison of gyrovirus DNA levels in 12 gyrovirus-positive fecal samples. The viral load of gyrovirus DNA were determined by quantitative PCR assay and calculated by normalization to the standard curve. The copy numbers were presented as the means from three repeated reactions per sample.

Between January 2016 and November 2017, a total of 197 fecal samples were collected from Jinzhou (n = 17), Shenyang (n = 36), Changchun (n = 85), Jilin (n = 33) and Harbin (n = 26) in northeast China. According to the PCR-based detection, twelve fecal samples, including one collected from Shenyang and Harbin each, two from Jilin and eight from Changchun, were positive for gyrovirus, with an apparent prevalence of 6.1% (12/197). Of these gyrovirus-positive samples, four fecal samples were positive for CAV, three were positive for HGyV/AGV2, three were positive for GyV3 and two were positive for GyV6, respectively (Table 1). One CAV-positive sample, two GyV3-positive samples and one GyV6-positive sample were detected from domestic cats with diarrhea, other eight gyrovirus-positive samples were from healthy cats. Healthy cats had a high gyrovirus positive rate (8.7%, 8/92) than diarrhoeic cats (3.8%, 4/105). We also detected the chicken DNA in 12 gyrovirus-positive feces, and found the chicken DNA in two CAV-positive samples (17JL0310 and 17SY0902) and one HGyV/AGV2-positive sample (16CC1103) (Table 1). These three fecal samples were collected from healthy cats. Copy numbers in gyrovirus-positive cats ranged 62,483 copies/ml to 1,317,593 copies/ml (Table 1). For CAV and HGyV/AGV2 positive samples, the viral loads were higher in fecal samples which tested to be positive for chicken DNA. While the viral copy numbers in cats with diarrhea (391,652–1,317,593 copies/ml, mean 825,998 copies/ml) are higher than that in healthy cats (95,113–558,771 copies/ml, mean 326,942 copies/ml) for GyV3 and GyV6 positive samples (Fig. 1).

**Table 1:** Details of the 12 cat-origin gyrovirus sequences identified in this study.

Sample name	Year	Region	Healthy status	Gyrovirus Species	DNA copy numbers (per ml)	Detection results of chicken DNA	Accession No.
17CC0509	2017	Changchun	Normal	CAV	628,377	—	MK089240
17SY0902	2017	Shenyang	Diarrhea	CAV	743,119	+	MK089243
17JL0310	2017	Jilin	Normal	CAV	1,051,657	+	MK089241
17JL0314	2017	Jilin	Normal	CAV	517,110	—	MK089242
16CC1103	2016	Changchun	Normal	HGyV/AGV2	395,804	+	MK089245
17CC0315	2017	Changchun	Normal	HGyV/AGV2	62,483	—	MK089244
17CC0810	2017	Changchun	Normal	HGyV/AGV2	100,915	—	MK089246
17CC0704	2017	Changchun	Normal	GyV3	95,113	—	MK089247
17CC0711	2017	Changchun	Diarrhea	GyV3	391,652	—	MK089248
17HRB0905	2017	Harbin	Diarrhea	GyV3	768,749	—	MK089249
17CC0301	2017	Changchun	Normal	GyV6	558,771	—	MK089250
17CC1116	2017	Changchun	Diarrhea	GyV6	1,317,593	—	MK089251

### Genomic analysis and sequences identities of the 12 gyrovirus strains

**Figure 2:**
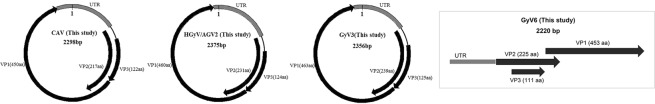
Comparative genomic organization of the cat-origin CAV, HGyV/AGV2, GyV3 and GyV6 identified in the present study. The genome sizes (nucleotides) and predicted sizes of viral protein (amino acids) were observed in the schematic diagram of genomes.

The complete cat-sourced gyrovirus genome sequences were verified using inverse PCR. Ten complete circular genome sequences (17CC0509, 17SY0902, 17JL0310, 17JL0314, 16CC1103, 17CC0315, 17CC0810, 17CC0704, 17CC0711 and 17HRB0905) and two near full-length genome sequences (17CC0301 and 17CC1116) were acquired and deposited in GenBank under accession numbers MK089240-MK089251. The length of the genome of 17CC0509, 17SY0902, 17JL0310 and 17JL0314 was 2298-bp, similar to the genome size of the CAV reference strains isolated from chickens, dogs and mice. Three partial overlapping ORFs, VP1 (450-aa), VP2 (217-aa) and VP3 (122-aa), were identified in these four CAV strains (Fig. 2). Comparative genomic analysis showed that the higher nucleotide identities were found between the four sequences and 22 CAV sequences retried from GenBank, with a mean value of 97.4% (Table 2). 17JL0310, 17JL0314 and 17SY0902 shared the highest nucleotide identities with CAV strains SH11 (99.8%, accession no. DQ141670), HN9 (99.1%, accession no. DQ141672) and 98D02152 (99.4%, accession no. AF311892) isolated from chicken, whereas 17CC0509 displayed the highest pairwise nucleotide identity (99.3%) with CAV isolated from human feces in Beijing, China (accession no. JQ690762). All four sequences displayed the lowest pairwise nucleotide identities (94.7–95.8%) with the CAV strain, 3711 (accession no. EF683159).

**Table 2:** Mean nucleotide percentage pairwise identity in full-length genome between the 12 cat-origin gyrovirus strains identified in the present study and reference strains from GenBank representing ten gyrovirus species.

	CAV^R^	HGyV/AGV2^R^	GyV3^R^	GyV4^R^	GyV5^R^	GyV6^R^	GyV7^R^	GyV8^R^	GyV9^R^	GyV10^R^
CAV^a^	97.4	47.7	50.8	28.1	25.4	48.2	46.4	36.4	43.1	42.9
HGyV/AGV2^b^	46.8	96.6	58.8	25.7	24.3	54.2	49.7	34.2	43.0	40.5
GyV3^c^	49.7	59.2	99.1	24.6	24.4	54.6	51.4	34.4	43.2	41.8
GyV6^d^	47.6	54.2	54.2	24.3	23.8	98.9	57.6	32.9	41.2	40.5

^a^Cat-origin CAV strains identified in this study: 17CC0509, 17SY0902, 17JL0310 and 17JL0314; ^b^Cat-origin HGyV/AGV2 strains identified in this study: 16CC1103, 17CC0315 and 17CC0810; ^c^Cat-origin GyV3 strains identified in this study: 17CC0704, 17CC0711 and 17HRB0905; ^d^Cat-origin GyV6 strains identified in this study: 17CC0301 and 17CC1116; ^R^Reference gyrovirus strains in different gyrovirus species retried from GenBank.

The genome of 16CC1103, 17CC0315 and 17CC0810 was 2375-bp long, encoding VP1 (460-aa), VP2 (231-aa) and VP3 (124-aa), respectively (Fig. 2). Compared to the gyrovirus reference sequences, the three strains were more closely related to the HGyV/AGV2 reference strains, with a mean nucleotide identity of 96.6% (Table 2). The strain 16CC1103 shared the highest nucleotide identity (99.7%) with Brazilian chicken-sourced AGV2 strain, Ave3 (accession no. HM590588), whilst it shared the lowest identity (95.0%) with AGV2 strain G17 (accession no. KJ452213) isolated from ferret fecal samples. Contrary to 16CC1103, 17CC0315 and 17CC0810 shared the highest nucleotide identities (99.2% and 98.9%) to the ferret-sourced HGyV/AGV2 strains G13 (accession no. KJ452214) and G17, respectively, while both of the two strains shared the lowest identities with Ave3 isolated from either a chicken in Brazil.

For 17CC0704, 17CC0711 and 17HRB0905, the length of the complete genome was 2356-bp, with three overlapping ORFs for the VP1 gene (1392-bp), VP2 gene (720-bp) and VP3 gene (378-bp) (Fig. 2). The three strains shared more than 99.1% nucleotide identities with GyV3 reference strains. And, more remarkable, 17CC0704 and 17CC0711 were more closely related to chicken-source GyV3 strain, SDAU-1 (accession no. MG366592), isolated in China, with the high nucleotide identities of 99.6–99.7%, whereas 17HRB0905 shared a higher identity of 99.5% to human-sourced GyV3 strain, FecGy (accession no. JQ308210).

Furthermore, the nearly complete genome sequences of 17CC0301 and 17CC1116, with length of 2220-bp, were acquired in the present study. Both 17CC0301 and 17CC1116 showed three overlapping ORFs, encoding VP1 (453-aa), VP2 (225-aa) and VP3 (111-aa) (Fig. 2), and showed the highest nucleotide identities (98.7% and 99.1%) with the GyV6 strain, Tu789 (accession no. KF294862), isolated from human feces in Tunisia in 2013.

### Phylogenetic analysis

**Figure 3:**
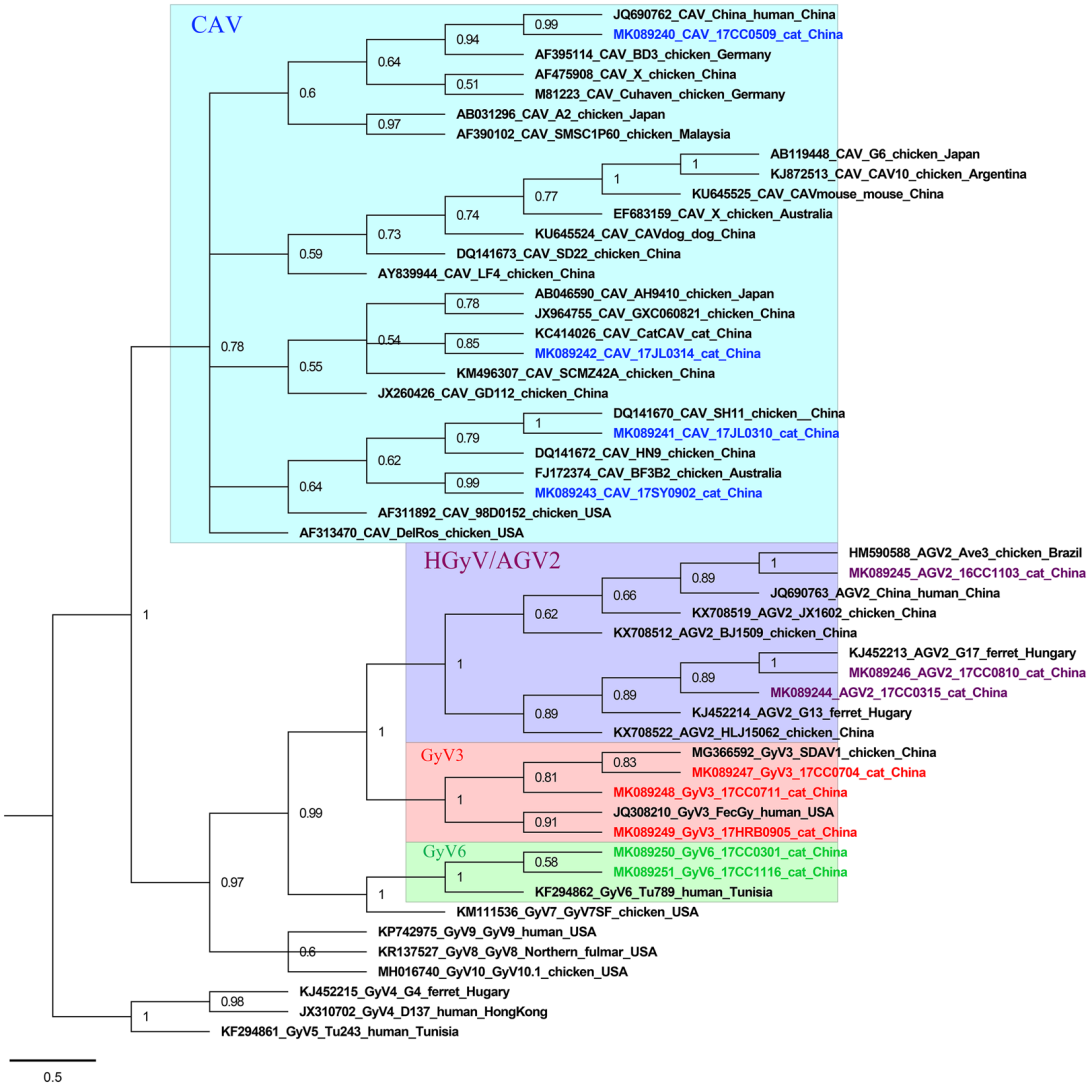
Bayesian phylogenetic tree constructed based on the whole genome nucleotide sequences of the 12 cat-origin gyroviruses and 40 reference gyroviruses. Branch lengths are measured in nucleotide substitutions. Numbers above branches indicate the posterior probability from the Bayesian approach. The tree is rooted at the midpoint of the longest branch. Cat-origin CAV, HGyV/AGV2, GyV3 and GyV6 strains identified in the present study are shown in blue, purple, red and green color, respectively.

A phylogenetic tree based on these 12 gyrovirus genome nucleotide sequences and 40 representative genome nucleotide sequences from ten gyrovirus species was constructed using different methods. The Bayesian tree showed that the 12 gyrovirus sequences were classified into different groups, supported by the topology and the high posterior probability (Fig. 3). 17CC0509, 17SY0902, 17JL0310 and 17JL0314 were placed within the group CAV, which included 23 CAV reference strains isolated from different hosts (including chicken, human, cat, dog and mouse); 16CC1103, 17CC0315 and 17CC0810 were more closely related to 7 HGyV/AGV2 reference strains identified in human, chicken and ferret, clustering within the group HGyV/AGV2; 17CC0704, 17CC0711 and 17HRB0905 were classified within the group GyV3 of which 17CC0704, 17CC0711 and chicken-sourced GyV3 strain SDAU-1 formed a small branch, while 17HRB0905 and human-sourced GyV3 strain FecGy were clustered within another branch; 17CC0301, 17CC1116 and GyV6 reference strain Tu789 cluster together, belonging the group GyV6. This classification was also supported by the ML phylogenetic analysis (Supplementary Fig. S1).

**Figure 4:**
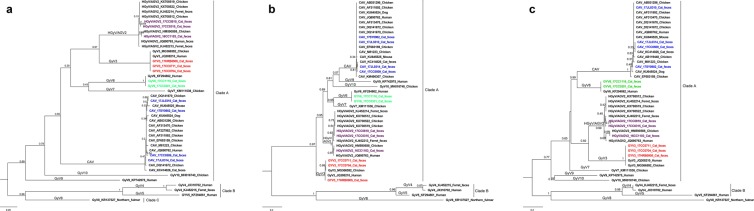
Phylogenetic analysis based on the VP1 (**a**), VP2 (**b**) and VP3 (**c**) amino acid sequences of cat-origin and reference gyroviruses using MrBayes. Branch lengths are measured in nucleotide substitutions. Numbers above branches indicate the posterior probability from the Bayesian approach. The tree is rooted at the midpoint of the longest branch. Cat-origin CAV, HGyV/AGV2, GyV3 and GyV6 strains identified in the present study are shown in blue, purple, red and green color, respectively. The gyrovirus species and phylogenetic clades were showed in the tree.

We then constructed the phylogenetic trees based on the deduced amino acid sequences of VP1, VP2 and VP3 genes using the Bayesian analysis with the JTT model (Fig. 4). The phylogenetic analyses based on VP1, VP2 and VP3 amino acid sequences showed the identical classification with that based on the complete genome sequences. The cat sourced CAV strain 17JL0310 shared 100% nucleotide and amino acid identities with the reference strain SH11 (accession no. DQ141670) in the VP2 and VP3 gene. 17JL0314 shared 99.7–99.8% nucleotide and 100% amino acid identities with reference strain isolated from feline feces in China (accession no. KC414026) in the VP2 and VP3 genes (Supplementary Table S4). 17JL0310 and 17SY0902 shared higher nucleotide and amino acid identities with the chicken-sourced CAV strain SH11 in the VP1, VP2 and VP3 genes. While 17CC0509 and 17JL0314 shared higher pairwise identities with human-sourced and cat-sourced CAV strains. In the Bayesian trees based on the VP1, VP2 and VP3 genes, all HGyV/AGV2 strains cluster within two distinct subgroups with >0.98 posterior probability. 16CC1103 shared the highest amino acid identity with the strain Ave3 (accession no.HM590588) isolated from chicken, while 17CC0315 and 17CC0810 were more closely related to the strain G17 (accession no.KJ452213) isolated from the feces of pet ferrets. All GyV3 strains identified in this study were 100% similar to GyV3 reference strains isolated from chicken meats and human feces based on the amino acid sequences of the VP1 gene, and shared 100% amino acid identities with GyV3 strain SDAV1 identified from chicken meats in the VP3 gene. The two cat-sourced GyV6 strains were 100% similar to GyV6 reference strain in both nucleotide and amino acid sequences of VP3 gene. Moreover, all of the gyroviruses could be further grouped into three distinct clades A, B and C in the Bayesian trees based on the amino acid sequences of VP1, VP2 and VP3 gene, similar to the previous reports. All cat-sourced gyrovirus strains belonged to the gyrovirus clade A.

We analyzed the recombination events for the 12 cat-origin gyrovirus strains using five methods in this study, but no recombination breakpoint was found in these strains.

### Molecular characterization

**Figure 5:**
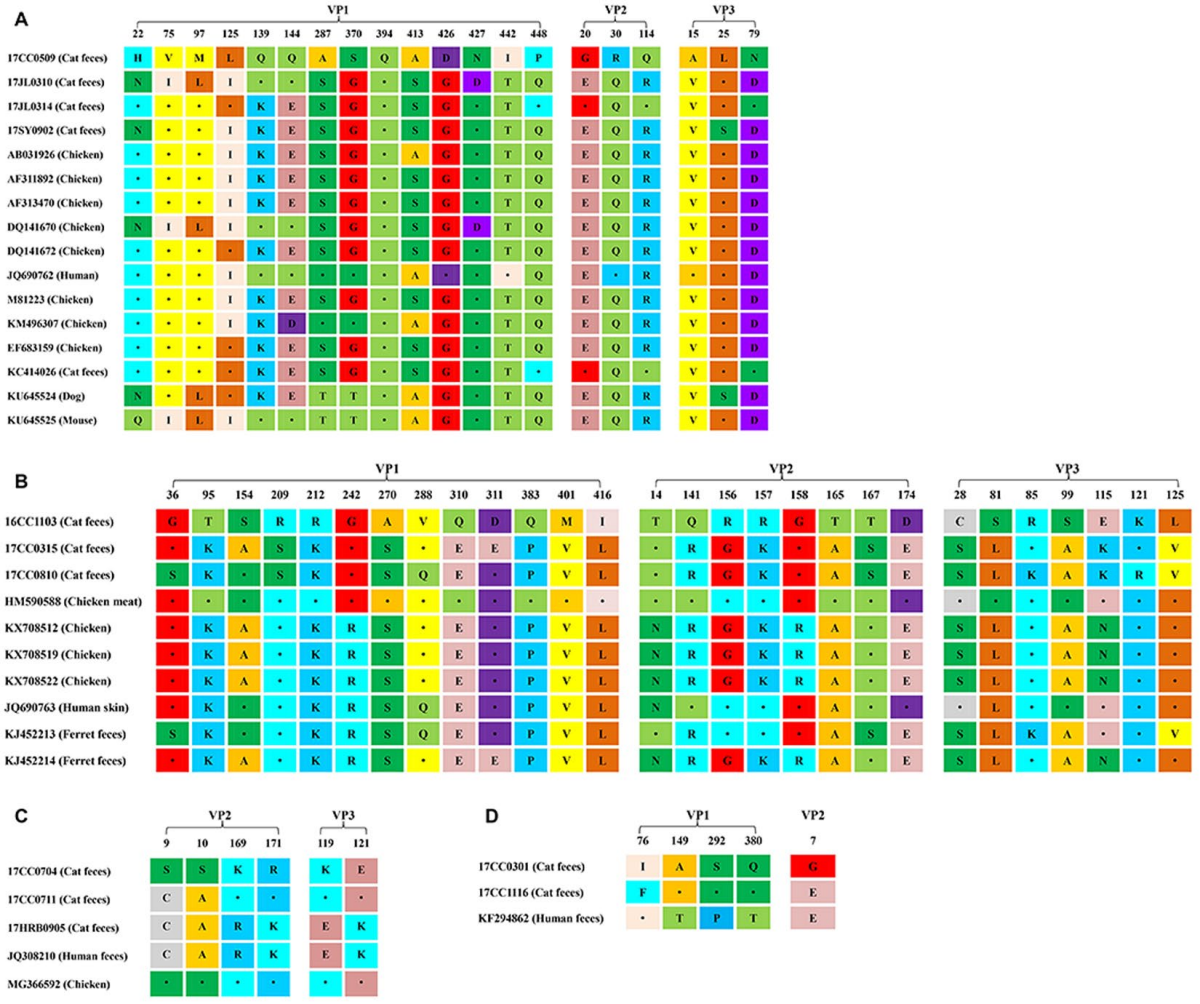
Amino acid polymorphisms and substitutions observed among the cat-origin CAV (**A**), HGyV/AGV2 (**B**), GyV3 (**C**) and GyV6 (**D**) strains identified in the present study.

To further analyze molecular characteristics of the 12 complete genome sequences of gyroviruses isolated from cats in this study, we systematically compared the amino acid polymorphisms of the 12 cat-sourced gyroviruses and representative gyrovirus strains isolated from different hosts (Fig. 5). The amino acid polymorphisms were found in the 19, 28, 6 and 5 amino acid sites in VP1-VP3 genes of cat feces-derived CAV, HGyV/AGV2, GyV3 and GyV6 strains, respectively. No amino acid substitutions were identified to be commonly possessed only by the cat feces-origin CAV and GyV3 isolates. A previous study had demonstrated that amino acid residues 139 and 144 of the CAV VP1 protein played an important role in viral growth and spread, as Gln139 and/or Gln144 were associated with a decreased rate of spread of CAV isolates^28^. In the 4 cat-sourced CAV isolates, the VP1 residues 139 and 144 of 17CC0509 and 17JL0310 were both glutamines, suggesting that the growth and spread rates of these two strains might be relatively low, while 17JL0314 and 17SY0902 carried amino acid residues carried Lys139 and Glu144 in the VP1 protein. It was also demonstrated that the position 394 in VP1 protein of CAV was a major genetic determinant of virulence, with glutamine (Glu) and histidine (His) representing high and low pathogenicity, respectively^2,3^. All of the cat-sourced CAV strains identified in the present study contained a glutamine at position 394, suggesting that they might be highly pathogenic. In all three cat-sourced HGyV/AGV2 isolates, the arginine (Arg) at position 209 of the VP1 gene was replaced by serine (Ser) in 17CC0315 and 17CC0810. Furthermore, there were four unique amino acid mutations (Ile76Phe, Thr149Gla, Pro292Ser and Thr380Gln) in VP1 and one mutation (Glu7Gly) in VP2 of cat-origin GyV6 isolates, which had not been previously reported. Three out of these five substitutions, including Thr149Gla, Pro292Ser and Thr380Gln, were simultaneously observed in the VP1 gene of 17CC0301 and 17CC1116.

### Viral isolation

Three cell lines (CRFK, Caco-2 and MDCC-MSB1) were co-cultured with gyrovirus-positive fecal supernatants for 4 days and passaged to the fifth generation. No CPE were observed in the cultured cells, and no gyrovirus DNA was detected in culture lysates and supernatant by qPCR.

## Discussion

The members of the genus *Gyrovirus*, which consists of the CAV prototype and nine other unofficially recognized species (HGyV/AGV2, GyV3, GyV4, GyV5, GyV6, GyV7, GyV8, GyV9 and GyV10), have been identified in chickens^10,11,17,18^, humans^8,15,16,19,22^, sea birds^21^, mice^25^, ferrets^24,29^, dogs^26^ and cats^25^. To date, there is only one report that CAV circulating in domestic cats in China^25^. Whether other gyrovirus species are circulating in cats is currently unknown. In this study, twelve gyrovirus strains were identified in the fecal samples of domestic cats that were collected from four cities (Shenyang, Jilin, Changchun and Harbin) in northeast China, with an apparent prevalence of 6.1% (12/197). The multiple alignment based on the full-length sequences of the gyrovirus genomes showed that the 12 cat-sourced gyroviruses includes 4 CAVs, 3 HGyV/AGV2 strains, 3 GyV3 strains and 2 GyV6 strains. To our knowledge, this study is the first report of HGyV/AGV2, GyV3 and GyV6 detection in domestic cats. So far only three gyrovirus species, including CAV, HGyV/AGV2 and GyV3 were reported in China. CAV was first isolated from sera of chickens in 1996^30^ and was first identified in pediatric fecal samples in 2012. HGyV/AGV2 was detected for the first time in chickens and healthy humans in China in 2015^12^. Moreover, GyV3 was first isolated from commercial broiler chickens in 2018^17^. Our investigation also provides for the first time the molecular evidence that GyV6 is circulating in China.

Previous studies had demonstrated that CAV can cause atrophy of bone marrow hematopoietic tissue and lymphoid tissues in young chickens, and lead to severe anemia and immunosuppression^31,32^. However, the potential of nine other gyrovirus species is unknown. Maggi, *et al*. found that HGyV may infect HIV-infected patients and organ transplant recipients^15^. Phan, *et al*. reported that the high positive rate of CAV and GyV3 in feces from diarrhoeic and healthy Chilean children^16^. GyV5 and GyV6 were also isolated from the feces of diarrhoeic children in Tunisia^19^. This evidence was inadequate to demonstrate that gyrovirus could cause immunosuppression and/or gastrointestinal disease in mammalians (e.g., humans). Our investigation showed that CAV, GyV3 and GyV6 were detected in domestic cats with or without diarrhea, while HGyV/AGV2 was only isolated from healthy cats. The positive rate of gyroviruses DNA in healthy cats (8.7%) was higher than that in diarrhoeic cats (3.8%), suggesting the gyrovirus may be non-pathogenic for domestic cats. However, for GyV3 and GyV6 positive samples, higher viral copy numbers (391,652–1,317,593 copies/ml, mean 825,998 copies/ml) were observed in cats with diarrhea, suggesting GyV3 and GyV6 may be related to the diarrhea of cats. More studies are needed to further investigate the pathogenic potential of different gyrovirus species in cats.

Comparison of the VP1-VP3 amino acid sequences between the 12 cat-origin gyrovirus strains and the homologous reference gyrovirus sequences indicated that the amino acid polymorphisms were existed in cat-origin gyroviruses, but no unique amino acid substitutions were found in cat-sourced CAV and GyV3 isolates. It had been demonstrated that the amino acid at position 394 of the CAV VP1 protein was a major genetic determinant of virulence, and the amino acids at position 139 and 144 of the CAV VP1 protein play an important role in viral growth and spread^3,28^. All of the CAV strains identified in this study possessed glutamine at position 394, suggesting that they might be highly pathogenic. 17CC0509 and 17JL0310 possessed both glutamines at the VP1 residues 139 and 144, which suggested these two CAV strains might have low growth and spread rates. Moreover, a unique amino acid mutation was found at position 209 of the VP1 gene in 17CC0315 and 17CC0810, and four unique amino acid mutations in VP1 and one mutation in VP2 were identified in three cat-sourced GyV6 strains. Whether these unique amino acid substitutions is due to the change in hosts or these mutations made gyrovirus easier to spread to different hosts is unclear and needs to be further studied. The amino acid polymorphisms analysis suggested that these cat-origin gyroviruses possessed considerable genetic diversity.

Genomic analysis showed that the cat-sourced gyroviruses have the same genome organization with the homologous reference gyrovirus strains, and shared more than 94.0% nucleotide pairwise identities to the homologous reference gyrovirus sequences from chickens, humans and pet ferrets. Phylogenetic analyses based on the complete gene sequences, VP1, VP2 and VP3 showed that all of the cat-sourced gyroviruses were divided into different gyrovirus species, clustering within gyrovirus clade A, which consists of CAV, HGyV/AGV2, GyV3, GyV6, GyV7, GyV9 and GyV10. In the gyrovirus clade A, CAV, HGyV/AGV2, GyV3, GyV7 and GyV10 had been reported to infect chickens; CAV, HGyV/AGV2, GyV3, GyV6 and GyV10 had been detected in human skin or feces. Interestingly, we found that some cat-sourced gyrovirus strains were more closely related to the reference strains isolated from chickens, while others shared higher nucleotide and amino acid identities with the homologous reference gyrovirus sequences identified in humans. These findings suggested that these diverse cat-origin gyroviruses might originated from gyrovirus-infected chickens or humans carrying gyroviruses via the fecal oral route, and domestic cats might be not the natural hosts of gyroviruses. In the present study, we detected the chicken DNA in 12 gyrovirus-positive feces, and found the chicken DNA in two CAV-positive samples (17JL0310 and 17SY0902) and one HGyV/AGV2-positive sample (16CC1103). Moreover, the three chicken-DNA-positive samples (395,804–1,051,657 copies/ml, mean 730,193 copies/ml) had higher viral copy numbers than chicken-DNA-negative samples (62,483–628,377 copies/ml, mean 327,221 copies/ml). Even more remarkably, 17JL0310, 17SY0902 and 16CC1103 shared higher nucleotide and amino acid identities with chicken-sourced gyrovirus strains, while other cat-sourced CAV and HGyV/AGV2 strains identified in chicken-DNA-negative samples were phylogenetically related to human/ferret-sourced gyroviruses. Chicken meat and chicken powder are usually used to make commercial food for cats. Domestic cats may be reservoirs for gyrovirus through ingest these commercial chicken foods from gyrovirus-infected chickens. This could be an explanation why the CAV and HGyV/AGV2, widely circulating in chickens, were identified in the feces of cats. Similarly, domestic cats could contact gyrovirus via the contact with gyrovirus-infected humans. This study reveals that domestic cats may be potential reservoir for gyroviruses, and raises the potential threat of gyrovirus to the health of domestic cats and other mammals.

Furthermore, to isolate the cat-origin gyroviruses, we selected three cell types originating from cats (CRFK cell line), humans (Caco-2 cell line) and chickens (MDCC-MSB1 cell line). Unfortunately, no gyrovirus replication was detected in any of these cell lines. To our knowledge, only CAV could be cultured in MDCC-MSB1 cell line^33^, while other attempts to isolate the other gyrovirus species have failed. Because of the failure of virus isolation, there is no evidence that the gyrovirus could replicate in domestic cats.

In conclusion, we identified 12 cat feces-derived gyrovirus strains, including 4 CAVs, 3 HGyV/AGV2 strains, 3 GyV3 strains and 2 GyV6 strains, and described the complete genome of the 12 gyroviruses. Our investigation demonstrates that the diverse gyrovirus species were circulating in domestic cats, and provides the first molecular evidence for the circulation of HGyV/AGV2, GyV3 and GyV6 in domestic cats. These cat-origin gyroviruses possessed considerable genetic diversity. This study also raises the possibility that domestic cats, as reservoirs for gyroviruses, may inadvertently disseminate viruses to other species, e.g., humans and chickens.

## Material and Methods

### Clinical specimens

A total of 197 fecal samples were collected from 105 diarrhoeic cats and 92 healthy cats in five cities, including Jinzhou, Shenyang, Changchun, Jilin and Harbin, in northeast China during 2016–2017. All samples were stored at −70 °C until further use.

### DNA extraction and gyrovirus detection

Individual fecal sample was homogenized in phosphate-buffered saline (PBS) at a concentration of approximately 0.5 g/ml using a vortex oscillator (Thermo, USA), and then were centrifuged at 10,000 × g for 10 min to collect the supernatant. Total DNA was extracted from 250 μl of supernatant for each sample using AxyPrep^TM^ Body Fluid Viral DNA/RNA Miniprep Kit (CORNING, China) according to the manufacturer’s instruction. Ten pairs of specific primers of which 6 pairs were described in previous papers^18,20,22,25^ and 4 pairs were designed using Primer 5.0 to detect different gryovirus species. The primer sequences and amplification conditions are shown in Supplementary Table S1. The sizes of amplicons were 535-bp for CAV, 293-bp for HGyV/AGV2, 412-bp for GyV3, 285-bp for GyV4, 475-bp for GyV5, 473-bp for GyV6, 224-bp for GyV7, 371-bp for GyV8, 1841-bp for GyV9 and 414-bp for GyV10. The amplified products were separated after electrophoresis on 1.5% agarose gels at 160 V for 20 min, and were visualized using a gel documentation system (Wealtec, USA). Furthermore, a conventional reverse transcription PCR (RT-PCR) with forward primer 5′-CTGAAGTACCCCATTGAACACG-3′ and the reverse primer 5′-ACAGGACTCCATACCCAAGAAAG-3′ targeting a 618-bp fragment of chicken β-actin gene was used to evaluate the chicken DNA in gyrovirus-positive fecal samples.

### Quantitative PCR for the quantification of the viral DNA load

To quantify viral load in fecal samples, quantitative PCR (qPCR) was performed with TB GreenTM Fast qPCR mix (Takara, Japan) and ABI 7500 Fast Real-time PCR system (Invitrogen, USA), with the forward and reverse primers for VP1 gene of CAV, HGyV/AGV2, GyV3 and GyV6 (Supplementary Table S2). A 25 μl PCR mixture was used for per reaction and contained 12.5 l of 2 × TB premix Ex TaqII, 20 pmol of forward primer, 20 pmol of reverse primer and 1 μg template DNA or 2 μl diluted standard DNA. DNA was amplified using the ABI 7500 Fast Real-time PCR system using the following procedure: 95 °C for 5 min, followed by 40 cycles of 95 °C for 5 s and 60 °C for 30 s. The standard curve was plotted from the results of parallel PCRs performed on ten-fold serial dilutions (10^7^–10^2^ copies/ml) of standard DNA. Reactions for each sample were performed in triplicate. DNA absolute quantities of gyrovirus in fecal samples were calculated by normalization to the standard curve, and were presented as the means from three repeated reactions per sample.

### Amplification, cloning and sequencing of the gyrovirus genomes

To further analyze the genomic characteristics, the full-length genomes of different gyrovirus species were amplified by inverse PCR (primers sequences were shown in Supplementary Table S3). Three pairs of primers were used to verify the CAV genome. The amplicons were 843-bp, 989-bp and 802-bp in length, respectively, to cover the entire CAV genome^26^. To map the HGyV/AGV2 genome, three primer pairs were used to amplify 733-bp, 981-bp and 802-bp fragments encompassing the entire genome^10^. For the amplification of the GyV3 genome, two primer pairs targeting the 431-bp and 2203-bp regions were used for inverse PCR^17^. Moreover, two pairs of specific primers were designed based on the published GyV6 sequences in GenBank (KF294862) to amplify the complete GyV6 genome. The amplified fragments were 1846-bp and 594-bp, respectively. The PCR amplification was carried out in a total volume of 50 μl of reaction mixture containing 25 μl of Premix Ex Taq (Takara, Japan), 2 μl of 10 pM forward and reverse primers, 5 μl of sample DNA and 16 μl of RNase free water using a thermal cycler (BIOER, China). The amplification programs were shown in Supplementary Table S3. All PCR products were analyzed by 1.0% agarose gel electrophoresis, and visualized using a gel documentation system (Wealtec, USA).

The amplification fragments with the expected sizes for each primer pair were purified using AxyPrep DNA gel Extraction Kit (CORNING, China), and cloned into a PMD-18T vector (Takara, China) according to the manufacturer’s instruction. Then, the ligated products were transformed into Trans-T1 competent cells (Transgen Biotech, China). Plasmid DNA was extracted using AxyPrep Plasmid Miniprep kit (CORNING, China), and was sent to Sangon Biotech (Shanghai, China) for Sanger sequencing. Cloning and sequencing were performed at least three times for each PCR fragment of each sample.

### Genomic, phylogenetic and recombination analyses

The nucleotide sequences of the gyrovirus-positive samples were assembled using Seqman program in Lasergene 7.0 software package (DNASTAR Inc., Wisconsin). The ORFs of the complete gyrovirus genome were predicted using ORF finder (https://www.ncbi.nlm.nih.gov/orffinder/) and were further verified using Gene Marks (http://opal.biology.gatech.edu/GeneMark/genemarks.cgi). Multiple sequences alignment between the gyrovirus genomes identified in this study and the gyrovirus reference sequences from different hosts were performed using Clustal W method. Percentage pairwise identities based on the nucleotide and the deduced amino acid sequences were calculated using Bioedit and SDT^34^. Phylogenetic analysis of the 12 cat-sourced gyroviruses and 40 reference sequences obtained from GenBank was performed using different methods. Maximun likelihood (ML) analysis was performedi in MEGA7.0 using the Kimura 2-parameter substitution model, and the branch robustness was evaluated by a bootstrap of 1,000 replicates. Additionally, the Bayesian trees based on the amino acid sequences of VP1, VP2 and VP3, and the genome nucleotide sequences were conducted using the Jones Taylor Thornton (JTT) amino acid substitution model and General Time Reversible (GTR) nucleotide substitution model, respectively. Four independent chains were run for 20 million steps with trees and parameters sampled every 2000 steps, and the first 10% were removed as burn-in. Two GyV4 strains (accession no. KJ452215 and JX310702) and one GyV5 strain (accession no. KF294861) were used as outgroup sequences. The trees were visualized using Figtree v1.4.3 and annotated with the support of the posterior probability from the Bayesian approach.

Recombination analysis was performed using GENECONV, BootAcan, RDP, MaxChi and SiScan methods in Recombination Detection Program (RDP) 4.0 software to identify putative parental sequences with significance set at *P* values < 0.01 and the sliding window size set as 30 bp. Only recombination event supported by no fewer than three independent methods were regarded as positive. Then the putative recombination events were further identified using SimPlot analysis in SimPlot 3.5 software.

### Virus isolation

The fecal supernatants of gyrovirus-positive samples were filtered through a 0.22-μm membrane (Millipore, Ireland) for virus isolation. Then, 500 μl of the filtrate were inoculated onto CRFK (originating from cats), Caco-2 (originating from humans) and MDCC-MSB1 (originating from chicken) cells (approximate 1 × 10^7^ cells per culture flask). Three repetitions were set for each sample on the same cell line. After incubating for 1 hour, the inocula were removed and replaced with MEM which supplemented with 2% foetal calf serum, and continued incubating at 37 °C with 5% CO_2_. Cultures were directly inspected daily by microscopy for cytopathic effects (CPE) until 4 days postinoculation. The cultures were frozen and used for further passage. Passaging did not stop until the fifth generation, after which the sample was considered negative if no CPE was observed. Culture lysates and supernatant were also collected for gyrovirus qPCR.

### Ethics statement

Written informed consent was obtained from all pets’ owners before collecting fecal samples in this study. Sample collection was performed by veterinary assistant of our lab according to the approved guidelines and related regulations. All of the experimental procedures were reviewed and approved by the Ethics Committee and Institutional Review Board of Use Committee of Jilin Agricultural University.

## Supplementary information

supplementary information

## Data Availability

The whole genomes of the identified viruses in the present study were summited to the GenBank database under accession numbers of MK089240-MK089251.
