# Supplementary material for: Genomic Characterization of Diverse Gyroviruses Identified in the Feces of Domestic Cats
Source: Sci Rep. 2019 Sep 16;9:13303. doi: 10.1038/s41598-019-49955-8 (PMC6746754; doi:10.1038/s41598-019-49955-8)
Supplement: Supplementary file 1 — supplementary information [file 41598_2019_49955_MOESM1_ESM.pdf]

Genomic Characterization of Diverse Gyroviruses Identified in the Feces of Domestic Cats

Jiang-Ting Niu[1,+], Shu-Shuai Yi[1,+], Guo-Ying Dong[2], Yan-Bing Guo[1, 3], Yan-Li Zhao[4], Hai-Long Huang[1], Kai Wang[1], Gui-Xue Hu[1,*] & Hao Dong[5]

[1] College of Animal Science and Technology, Jilin Agricultural University, Changchun, China.

[2] College of Global Change and Earth System Science, Beijing Normal University, Beijing, China.

[3] Jilin Institute of Animal Husbandry and Veterinary Science, Changchun, China.

[4] Library, Jilin Agricultural University, Changchun, China.

[5] College of life Science, Jilin Agricultural University, Changchun, China.

* Correspondence and requests for materials should be addressed to G.X.H. (email: huguixue901103@163.com and huguixue901103@126.com)

[+] These authors contributed equally to this work.

Supplementary Table S1. Oligonucleotide sequences and amplification conditions of primers used for the detection of gyroviruses.

**Virus**	**Nucleotide sequence (3')**	**Amplicon size (bp)**	**Amplification program**
CAV	ATCAACGGTGTTCAGGC	535	94℃ for 5 min, followed by 35 cycles of 94℃ for 30 s, 57℃ for 30 s and 72℃ for 45 s, and a final extension at 72 ℃ for 10 min
	CCTTGGAAGCGGATAGTCAT		
HGyV/AGV2	CGCGTAGAAGATCCTTTGATC	293	94℃ for 5 min, followed by 35 cycles of 94℃ for 30 s, 52℃ for 30 s and 72℃ for 30 s, and a final extension at 72 ℃ for 10 min
	CTGCAAGTGCTGAGGATAAGG		
GyV3	GACACAGACTGCGACGAAGA	412	94℃ for 5 min, followed by 35 cycles of 94℃ for 30 s, 52℃ for 30 s and 72℃ for 30 s, and a final extension at 72 ℃ for 10 min
	ATGCTCCTGGCTGTCTAGAT		
GyV4	GTGGTATCGAAGTGGAAAGTACC	285	94℃ for 5 min, followed by 35 cycles of 94℃ for 30 s, 48℃ for 30 s and 72℃ for 30 s, and a final extension at 72℃ for 10 min
	CCCCCTGATACATACTGTACATA		
GyV5	CCGTGGGGGAGGCTTTACAA	475	94℃ for 5 min, followed by 35 cycles of 94℃ for 30 s, 58℃ for 30 s and 72℃ for 30 s, and a final extension at 72℃ for 10 min
	GCACCGCCATCCCAAACAC		
GyV6	CTTCCGCGACCACAAACCAC	473	94℃ for 5 min, followed by 35 cycles of 94℃ for 30 s, 56℃ for 30 s and 72℃ for 30 s, and a final extension at 72℃ for 10 min
	GCGGCCATTATAGCGACCTTAG		
GyV7	AGGATGGCAACTTTTCAGGC	224	94℃ for 5 min, followed by 35 cycles of 94℃ for 30 s, 50℃ for 30 s and 72℃ for 30 s, and a final extension at 72℃ for 10 min
	TGGCTGTGCCGGCCAGTAGT		
GyV8	GCGCCGCCATTACAGAGG	371	94℃ for 5 min, followed by 35 cycles of 94℃ for 30 s, 55℃ for 30 s and 72℃ for 30 s, and a final extension at 72℃ for 10 min
	CTTTTTGCGGCATATTAGTCCA		
GyV9	TGAAACACTTATTGGAGAACTGTACCT	1841	94℃ for 5 min, followed by 35 cycles of 94℃ for 30 s, 62℃ for 30 s and 72℃ for 2 min, and a final extension at 72℃ for 10 min
	TTGTCCTCTCTTTGAGCCTCTGTC		
GyV10	CCCCGGCTCGTATGTGGTAAGA	414	94℃ for 5 min, followed by 35 cycles of 94℃ for 30 s, 60℃ for 30 s and 72℃ for 30 s, and a final extension at 72℃ for 10 min
	GGCTGTGCGGGGAAATAGTCAA		

Supplementary Table S2. Oligonucleotide sequences of specific primers used for quantitative PCR.

Virus	Nucleotide sequence (5'-3')	Amplicon size (bp)
CAV	AGCGCAAGGCACAAATAAGTC	90
	GCCCATGAGTCGCTCTTCA	
HGyV/AGV2	TGGTGCGTGGTGAAAGTC	96
	CGTTGCAGTGTGCTCGTTGT	
GyV3	TCCCACAACACATCCAGG	140
	AAGAAGAAGTGCCCATCG	
GyV6	TTCTCAGCGTTATCAGCCCTT	167
	GTGTCATCTGTAATCCATTCG	

Supplementary Table S3. Primers used for the gyrovirus genome amplification.

Virus	Nucleotide sequence (5'-3')	Amplicon size (bp)	Amplification program
CAV	GCATTCCGAGTGGTTACTATTCC	843	95℃ for 5 min, followed by 30 cycles of 95℃ for 30 s, 55℃ for 50 s and 72℃ for 60 s, and a final extension at 72 ℃for 10 min
	CGTCTTGCCATCTTACAGTCTTAT		
	CGAGTACAGGGTAAGCGAGCTAAA	989	95℃ for 5 min, followed by 30 cycles of 95℃ for 30 s, 55℃ for 50 s and 72℃ for 60 s, and a final extension at 72 ℃for 10 min
	TGCTATTCATGCAGCGGACTT		
	ACGAGCAACAGTACCCTGCTAT	802	95℃ for 5 min, followed by 30 cycles of 95℃ for 30 s, 55℃ for 50 s and 72℃ for 60 s, and a final extension at 72 ℃for 10 min
	CTGTACATGCTCCACTCGTT		
HGyV/AGV2	TCACAGCCAATCAGAATTGAGCACG	733	95℃ for 5 min, followed by 30 cycles of 95℃ for 30 s, 60℃ for 50 s and 72℃ for 60 s, and a final extension at 72 ℃for 10 min
	TTCTACGCGCATATCGAAATTTACC		
	TATTCCCGGAGGGGTAAATTTCGAT	981	95℃ for 5 min, followed by 30 cycles of 95℃ for 30 s, 60℃ for 50 s and 72℃ for 60 s, and a final extension at 72 ℃for 10 min
	CCCCTGTCCCCGTGATGGAATGTTT		
	ATTTCCTAGCACTCAAAAACCCATT	802	95℃ for 5 min, followed by 30 cycles of 95℃ for 30 s, 60℃ for 50 s and 72℃ for 60 s, and a final extension at 72 ℃for 10 min
	TCTGGGCGTGCTCAATTCTGATT		
GyV3	GACACAGACTGCGACGAAGA	431	95℃ for 5 min, followed by 30 cycles of 95℃ for 30 s, 60℃ for 50 s and 72℃ for 30 s, and a final extension at 72 ℃for 10 min
	ATGCTCCTGGCTGTCTAGAT		
	AAGGTCAAAGATTGGAGGAC	2203	95℃ for 5 min, followed by 30 cycles of 95℃ for 30 s, 60℃ for 60 s and 72℃ for 2 min, and a final extension at 72 ℃for 10 min
	TAGGTATGAATACGAGCC		
GyV6	TACTTACGCGCAGCAGATCGT	1846	95℃ for 5 min, followed by 30 cycles of 95℃ for 30 s, 65℃ for 60 s and 72℃ for 2 min, and a final extension at 72 ℃for 10 min
	CGCTCCAAGGGCTGATAACG		
	AATGACAGGCATGATACGC	594	95℃ for 5 min, followed by 30 cycles of 95℃ for 30 s, 55℃ for 50 s and 72℃ for 30 s, and a final extension at 72 ℃for 10 min
	CCCATTTACCAATCAGCT		

Supplementary Table S4. Percentage pairwise sequences identities (nucleotide/amino acid) among cat-origin gyroviruses and representative reference strains. The highest nucleotide and amino acid pairwise identities are shown in red color, the lowest are shown in blue color.

Isolate	Representative gyrovirus strains	Genome	VP1	VP2	VP3
**CAV**					
17CC0509	JQ690762 (China/Human/China)	99.3/-	99.3/99.3	99.7/99.1	99.7/99.2
	KC414026 (CatCAV/Cat/China)	98.1/-	97.9/98.0	99.4/99.1	99.5/99.2
	DQ141670 (SH11/Chicken/China)	97.6/-	97.6/97.3	98.9/98.6	99.2/98.4
	KU645524 (CAVdog/Dog/China)	96.3/-	95.2/96.6	98.6/98.6	98.6/96.8
	KU645525 (CAVmouse/Mouse/China)	95.8/-	94.0/97.0	98.4/97.2	99.2/97.6
	EF683159 (X/Chicken/Australia)	94.7/-	93.9/98.0	98.4/98.1	98.1/95.1
17JL0310	JQ690762 (China/Human/China)	98.0/-	98.1/97.8	99.2/99.5	99.5/99.2
	KC414026 (CatCAV/Cat/China)	98.2/-	97.9/97.1	99.2/98.6	99.2/99.2
	DQ141670 (SH11/Chicken/China)	99.8/-	99.9/99.8	100/100	100/100
	KU645524 (CAVdog/Dog/China)	97.2/-	96.0/96.9	99.7/100	99.5/98.4
	KU645525 (CAVmouse/Mouse/China)	96.6/-	95.0/98.0	98.9/98.6	99.5/99.2
	EF683159 (X/Chicken/Australia)	95.5/-	94.8/98.7	99.1/99.5	98.9/96.7
17JL0314	JQ690762 (China/Human/China)	97.3/-	97.4/97.8	98.9/98.2	98.9/98.4
	KC414026 (CatCAV/Cat/China)	99.1/-	99.1/98.9	99.8/100	99.7/100
	DQ141670 (SH11/Chicken/China)	98.6/-	98.2/98.0	99.4/98.6	99.5/99.2
	KU645524 (CAVdog/Dog/China)	97.1/-	96.2/97.3	99.1/98.6	98.9/97.5
	KU645525 (CAVmouse/Mouse/China)	96.0/-	94.3/96.9	98.6/97.2	98.9/98.4
	EF683159 (X/Chicken/Australia)	95.3/-	94.8/99.6	98.8/98.2	98.4/95.9
17SY0902	JQ690762 (China/Human/China)	97.9/-	98.0/98.0	99.1/99.5	99.2/98.4
	KC414026 (CatCAV/Cat/China)	98.5/-	98.4/98.2	99.1/98.6	98.9/98.4
	DQ141670 (SH11/Chicken/China)	99.0/-	99.0/98.7	99.8/100	99.7/99.2
	KU645524 (CAVdog/Dog/China)	97.5/-	96.4/97.6	99.6/99.8	99.5/99.2
	KU645525 (CAVmouse/Mouse/China)	96.8/-	95.3/97.3	98.8/98.6	99.2/98.4
	EF683159 (X/Chicken/Australia)	95.8/-	95.3/96.8	98.9/99.5	98.6/95.9
**HGyV/AGV2**					
16CC1103	JQ690763 (China/Human/China)	98.0/-	97.5/97.9	99.1/99.1	98.7/96.8
	KJ452213 (G17/Ferret/Hungary)	95.0/-	93.0/97.4	97.1/95.7	96.3/92.0
	KJ452214 (G13/Ferret/Hungary)	95.7/-	94.5/97.8	97.3/97.0	96.3/93.6
	HM590588 (Ave3/Chicken/Brazil)	99.7/-	99.9/100	99.9/100	99.7/99.2
17CC0315	JQ690763 (China/Human/China)	95.7/-	94.6/98.7	97.7/96.1	97.1/94.4
	KJ452213 (G17/Ferret/Hungary)	99.2/-	99.1/99.6	99.3/99.1	98.9/96.8
	KJ452214 (G13/Ferret/Hungary)	95.8/-	94.4/98.3	98.6/98.7	97.9/94.4
	HM590588 (Ave3/Chicken/Brazil)	95.6/-	94.1/97.8	97.6/96.1	96.5/93.6
17CC0810	JQ690763 (China/Human/China)	96.0/-	95.2/99.1	97.3/96.1	96.8/93.6
	KJ452213 (G17/Ferret/Hungary)	98.9/-	99.3/99.6	99.3/99.1	98.7/95.2
	KJ452214 (G13/Ferret/Hungary)	96.0/-	93.9/98.3	98.6/98.7	98.1/94.4

	HM590588 (Ave3/Chicken/Brazil)	**94.8**/-	**93.1/97.4**	**97.1/96.1**	**96.3/92.8**
**GyV3**					
17CC0704	JQ308210 (FecGy/Human/USA)	**98.7**/-	**98.6**/**100**	**99.2/98.3**	**99.5/98.4**
	MG366592 (SDAV1/Chicken/China)	**99.7**/-	**100/100**	**99.9/100**	**100/100**
17CC0711	JQ308210 (FecGy/Human/USA)	**98.8**/-	**98.6**/**100**	**99.4**/**99.2**	**99.5/98.4**
	MG366592 (SDAV1/Chicken/China)	**99.6**/-	**100/100**	**99.6/99.2**	**100/100**
17HRB0905	JQ308210 (FecGy/Human/USA)	**99.5**/-	**100/100**	**100/100**	**99.5/98.4**
	MG366592 (SDAV1/Chicken/China)	**98.9**/-	**98.6**/**100**	**99.0/98.3**	**100/100**
**GyV6**					
17CC0301	KF294862 (Tu789/Human/Tunisia)	98.7/-	99.1/99.3	99.7/99.6	100/100
17CC1116	KF294862 (Tu789/Human/Tunisia)	99.1/-	99.1/99.3	99.9/100	100/100

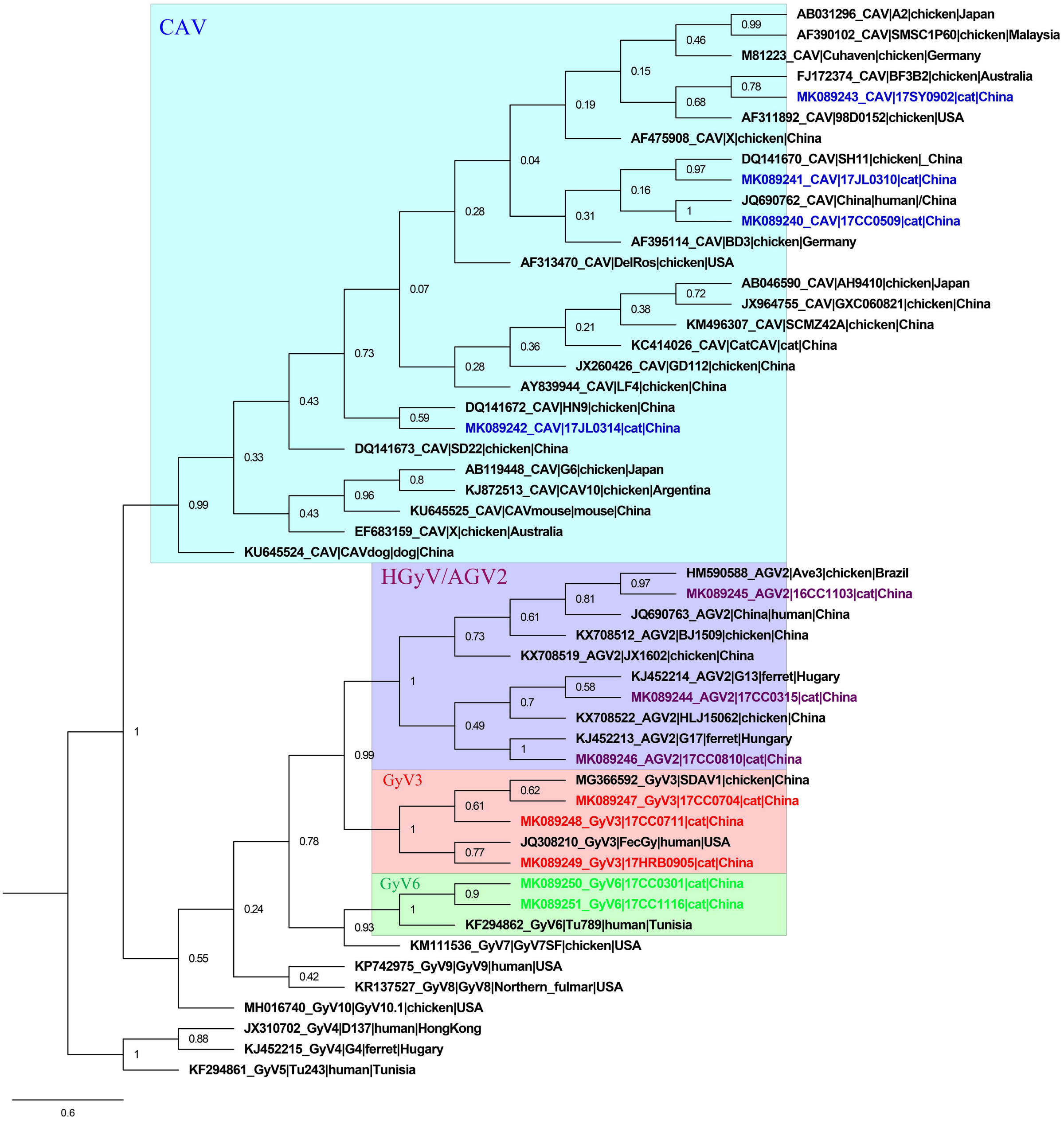

Supplementary Figure S1. Maximun likelihood phylogenetic tree based on the whole genome nucleotide sequences of the 12 cat-origin gyroviruses and 40 reference gyroviruses. Branch lengths are measured in nucleotide substitutions and numbers show branching percentages in bootstrap replicates. The tree is rooted at the midpoint of the longest branch. Cat-origin CAV, HGyV/AGV2, GyV3 and GyV6 strains identified in the present study are shown in blue, purple, red and green color, respectively.
